# Postpartum Hepatitis C Linkage to Care Program in a Co-located Substance Use Disorders Treatment Model

**DOI:** 10.1007/s10995-023-03770-w

**Published:** 2023-09-28

**Authors:** Aneesha Cheedalla, Katherine Hinely, Lauren Roby, O. Trent Hall, Carlos Malvestutto, Kara M. Rood

**Affiliations:** 1 Department of Obstetrics and Gynecology, McCampbell Hall, Ohio State University Wexner Medical Center, Columbus, OH USA; 2 Department of Psychiatry and Behavioral Health, Talbot Hall, Ohio State University Wexner Medical Center, Columbus, OH USA; 3 Department of Infectious Diseases, McCampbell Hall, Ohio State University Wexner Medical Center, Columbus, OH USA

**Keywords:** Hepatitis C, Postpartum, Linkage to care, Treatment, Opioid use disorder

## Abstract

**Purpose:**

Hepatitis C virus (HCV) is increasingly prevalent in pregnancy and among people with substance use disorders (SUD). Highly effective treatments are now available for chronic HCV. Qualifying for HCV treatment often requires preauthorization and several clinical criteria, including laboratory assessment of liver function and other infectious diseases and liver imaging to assess for fibrosis. Linkage to care (LTC) models have been shown to assist with obtaining the necessary clinical information (laboratory assessment/liver imaging) and improving HCV treatment rates in non-pregnant individuals.

**Description:**

Beginning in December 2020, a specialized LTC team identified patients with HCV viremia who were interested in postpartum treatment. The LTC team assisted patients with completing the necessary clinical criteria (laboratory assessment and liver imaging) for HCV treatment. Patients were then linked to infectious disease specialists who prescribed treatment to patients via telemedicine. Most patients identified with HCV were enrolled in our institution’s co-located obstetric and SUD program, which provides continued care until 1 year postpartum.

**Assessment:**

In 2019, an internal review identified that none of the 26 pregnant patients with HCV viremia in our co-located obstetric and SUD program were prescribed direct-acting antiviral (DAA) treatment within 12 months postpartum. Between December 2020 and July 2022, our HCV LTC team identified 34 patients with HCV who were eligible for treatment. Of these patients, 55% (19/34) obtained all necessary laboratory and liver imaging requirements and 79% (15/19) were prescribed DAA treatment after a telehealth visit with an infectious disease specialist. All fifteen patients who were prescribed treatment participated in the co-located obstetric and SUD program. The largest barrier to obtaining treatment was completing the necessary laboratory and liver imaging requirements for prescribing DAA. Only one patient who did not receive care in our co-located obstetric and SUD program had completed the necessary laboratory and liver imaging requirements to proceed with treatment but did not follow up with the infectious disease specialist for DAA treatment.

**Conclusion:**

Our HCV LTC program was successful in treating postpartum patients for HCV if they participated in the co-located obstetric and SUD program at our institution. Creating a partnership with an infectious disease specialist and utilizing telemedicine were beneficial strategies to connect patients to treatment for HCV during the postpartum period.

**Supplementary Information:**

The online version contains supplementary material available at 10.1007/s10995-023-03770-w.

## Significance

Hepatitis C virus (HCV) is increasingly prevalent in the pregnant population and among individuals with SUD. Highly effective HCV treatments are available postpartum, however LTC is underutilized during prenatal and postpartum care. A LTC model involving a co-located obstetric and SUD program, partnership with a referral department, and telemedicine was effective at improving HCV treatment rates at our institution.

**Supplementary Information:**

The online version contains supplementary material available at 10.1007/s10995-023-03770-w.

## Purpose

Hepatitis C virus (HCV) affects approximately 2.4 to 2.7 million people in the United States and is one of the leading causes of cirrhosis and liver failure in the country (Denniston et al., 2014). Increasing prevalence is attributed to the opioid epidemic in the United States and the increase in intravenous drug use (Holtzman et al., 2021). Overall, it is estimated that approximately 76% of people enrolled in opioid treatment programs have a HCV infection, compared to 1.8% among the general US population (Zeremski et al., 2014). The prevalence of the disease is increasing in the United States despite the existence of highly effective and well tolerated therapies (Holtzman et al., 2021).

Direct-acting antiviral (DAA) medications have been shown to cure disease in 96–99% of patients with short courses (typically 8–12 weeks) and are well tolerated (Backus et al., 2018). HCV eradication through treatment has been shown to have benefits to morbidity, mortality and quality of life (Backus et al, 2018; van der Meer et al., 2012; Younossi et al., 2016). The American Association for the Study of Liver Diseases (AASLD) and Infectious Diseases Society of America (IDSA) recommend treating most people with HCV, including those with SUD (AASLD-IDSA HCV Guidance Panel, 2018). However, it is estimated that only 23% of patients with Medicaid insurance in the United States received treatment for HCV between 2017 and 2020 (Thompson, 2022). When first developed, DAA treatment cost upwards of $90,000 per patient, which led states to develop restrictions for treatment eligibility, such as sobriety requirements, liver fibrosis scores and medical subspecialist prescribing requirements (Nephew et al., 2022; Younossi et al., 2017). Despite evidence that people who inject drugs can adhere to current treatment regimens and achieve sustained virologic response at 12 months and recommendations by the AASLD that active or recent drug use or concern for reinfection is not a contraindication to HCV treatment, several state Medicaid programs continued to maintain these requirements until very recently.

The pregnant population has also seen an increase in opioid use disorders, estimated to have increased five-fold between 2000 to 2009 (Patrick et al., 2012). Under these circumstances, pregnant people are at increased risk of contracting HCV, with rates increasing by 161% from 2009 to 2017 (Rossi et al., 2020). Pregnant people can more easily access healthcare, due to the greater availability of insurance coverage during pregnancy and the postpartum period. DAA treatment has not been routinely prescribed during pregnancy due to concern for safety and limited available literature on DAA use during pregnancy (Bernstein et al., 2018).

The postpartum period may be an optimal time for patients to receive treatment for HCV while they are connected with the healthcare system and eligible for treatment. Despite this opportunity, postpartum patients diagnosed with HCV have low diagnostic evaluation and treatment rates (Bushman et al., 2021; Page et al., 2017).

For patients who face barriers to care, such as lacking transportation or childcare, or living in a rural area, telemedicine provides a more accessible method of treatment. Telemedicine is proven to be beneficial for obstetric care (Brown & DeNicola, 2020). It is being studied extensively in the postpartum period, especially for treatment of conditions such as postpartum depression and pregnancy-related hypertensive disorders. Telemedicine may also improve low attendance rates (approximately 50%) for postpartum appointments (Adams et al., 2023).

Telemedicine has been utilized for patients with HCV to improve treatment rates for underserved nonpregnant populations and could also improve HCV treatment rates in the postpartum population (Arora & Thornton, 2020). Therefore, our institution sought to improve rates of HCV treatment by creating a LTC program for pregnant and postpartum patients to receive treatment and ultimately be cured of HCV through a partnership with an infectious disease specialist via telemedicine. The aims of this analysis are to determine the effectiveness of the program and to identify continued barriers for obtaining HCV treatment in this patient population.

## Description

The Ohio State University Wexner Medical Center is a large academic tertiary care center located in central Ohio. Pregnant patients connect with our institution by receiving prenatal care at our academic medical clinic or local offices or presenting to the labor and delivery unit as new patients or as transfers from other hospitals in the region. Pregnant patients with SUD are referred to the Substance Abuse Treatment, Education and Prevention Program (STEPP), which consists of co-located high-risk obstetric care, infectious disease screening, medication for opioid use disorder (MOUD), weekly behavioral health therapy groups, educational sessions, individual counseling, psychiatry services and case management. Buprenorphine is the primary MOUD prescribed to STEPP patients. While enrolled in the program, patients can receive SUD care for 1 year postpartum, including weekly behavioral health groups and access to MOUD.

An internal review in 2019 noted that of the 26 patients in STEPP who were eligible for HCV treatment postpartum, none of them received treatment within 12 months after delivery, based on our previous system of referral to the hepatology department upon discharge from the hospital following delivery. Given these results, we developed a different model of care through a quality improvement project, with the goal of reducing barriers to obtaining HCV treatment for eligible postpartum patients.

**Fig. 1:**
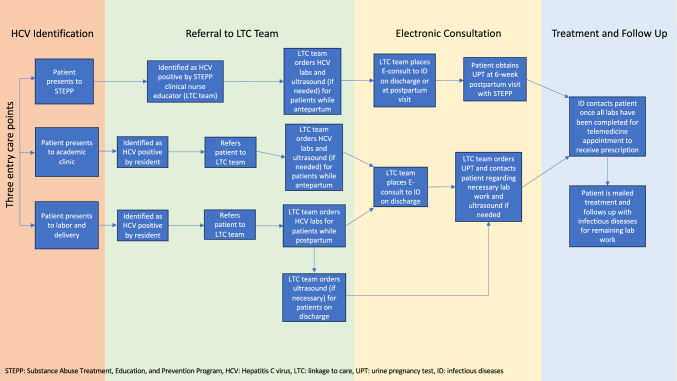
Process map for linkage to care model

Patients with HCV viremia (HCV RNA > 12 IU/mL) in STEPP, labor and delivery, or the academic medical clinic were identified and counseled regarding HCV treatment in the antepartum or postpartum period. Patient educational tools were developed and provided when discussing HCV treatments regarding safety with breastfeeding. The educational materials were distributed to all OB-GYN residents and staff (see Supplementary Material). Identified patients were then referred via electronic medical records to a LTC team. The team consisted of a resident, fellow, medical student, attending physician, and clinical nurse educator. The team assisted eligible and interested patients to receive the necessary laboratory assessments and liver imaging. Several insurance companies serving Medicaid patients require assessment of liver fibrosis/cirrhosis via imaging (either a Fibroscan, MRI with elastography protocol or a specific liver elastography ultrasound) prior to treatment. Once the patient was in the postpartum period, an electronic consult from the LTC team was sent to the infectious diseases department. The LTC team then assisted patients with completing any additional labs or imaging that were identified with the electronic consult and labs that could not be done immediately postpartum, such as urine pregnancy tests. Once patients completed the necessary steps, they were scheduled for a telemedicine visit with an infectious disease specialist, who reviewed their candidacy and prescribed a DAA treatment regimen if indicated. Medicaid covered the cost of the medications for all patients who performed the appropriate prerequisite tests. The process to obtain treatment is detailed in Fig. 1.

The team followed up with patients monthly to ensure all steps were completed in order to access treatment. Patients were contacted three times, and if unable to reach them, a letter was sent to the patient with further instructions should they be able to complete the steps for HCV treatment.

We assessed the number of patients who were identified for treatment; obtained serum labs, a urine pregnancy test and electronic consult to the infectious diseases department; prescribed and completed treatment with documented sustained virologic response. Patient characteristics of maternal age, race, ethnicity, prenatal care, active substance use and receiving MOUD were collected. Active drug use was defined as a positive urine drug screen on presentation to the labor and delivery unit and prior to receiving any analgesic medication. If a urine drug screen was not available during the labor and delivery admission, the most recent drug screen obtained prior to admission was utilized. Scant prenatal care was identified as less than three prenatal visits during the pregnancy. MOUD was identified as any patient receiving a prescription for buprenorphine or methadone during the pregnancy. Patients identified from outside providers were those who presented as transfers to the institution for delivery.

## Assessment

Between December 2020 and July 2022, 34 patients were identified to have HCV viremia and referred for treatment through our LTC program. Patient demographics are noted in Table 1. Of the patients identified and referred, 22 were STEPP patients, five patients had outside prenatal care providers, and four patients had scant or no prenatal care. Nine patients had active drug use, and two of these patients with active use had prenatal care with STEPP and received MOUD, while the remaining patients had scant care or received care at an outside hospital (Table 1). The patients with active drug use were identified following the removal of sobriety requirements for treatment, thus were deemed eligible for treatment.

**Table 1:** Patient demographics of postpartum patients identified for Hepatitis C treatment

Characteristics	N
Maternal age (range in years)	31 (23–40)
Race (%)	
White	34 (100)
Ethnicity (*%*)	
Non-Hispanic or Latino	34 (100)
Prenatal care *(%)*	
Multidisciplinary SUD treatment	22 (65)
OB clinic	3 (9)
Outside prenatal care	5 (15)
Prenatal care scant or none	4 (12)
Active substance use^a^ (%)	9 (26)
Received MOUD (%)	24 (71)

Sample N = 34

*SUD* substance use disorder, *MOUD* medications for opioid use disorder (buprenorphine, buprenorphine/naloxone, methadone)

^a^Active substance use defined as a positive urine drug screen on presentation to labor and delivery and prior to receiving any analgesic medication

Overall, 24 out of the 34 patients obtained the necessary serum labs for treatment and 19 patients obtained a urine pregnancy test. Fifteen patients were prescribed treatment. Twelve patients completed treatment and have documented sustained virologic response thus far, while three patients are currently in the process of completing treatment. All fifteen patients who were prescribed HCV treatment participated in STEPP. Two patients showed spontaneous clearance of HCV on follow up labs, thus did not require treatment. Of the 17 out of 32 patients who have not started or completed treatment, two patients did not obtain a telemedicine appointment with the infectious diseases department, four patients are awaiting electronic consult recommendations with the infectious disease specialist, eight patients needed further labs or were unable to be contacted to obtain labs, one patient was referred to the gastroenterology department and has an appointment scheduled, one patient desired treatment closer to their residence, and one patient become pregnant prior to initiation of treatment. Eight out of the nine patients who received scant prenatal care or care at an outside hospital did not obtain the necessary labs for treatment and were unable to be contacted.

## Conclusions

Our LTC program for HCV in a co-located obstetric and SUD program was successful in increasing the number of postpartum patients receiving HCV treatment at our institution. 44% (15/34) of postpartum patients have received treatment for HCV thus far, compared to zero patients in the prior year. Patients who participated in STEPP, including postpartum care, had much higher rates of completion compared to those who received prenatal care elsewhere or received no prenatal care. A partnership between the infectious diseases department and a lead physician to determine if patients met the criteria for treatment also played a crucial role in the success of the program.

Ohio and other states in the Appalachia region have a high prevalence of HCV infections and perinatal transmission (2020 Number of Newly Reported Perinatal Hepatitis C | CDC, 2022; Rosenberg et al., 2018). Our program utilizes the micro-elimination approach that is recommended for vulnerable populations at high risk for HCV to decrease disease prevalence in high-risk populations (Hollande et al., 2020), and may ultimately decrease rates of vertical transmission with future pregnancies if implemented on a larger scale.

The postpartum period has been shown to have a high risk of overdose, with the highest rates occurring 7–12 months after delivery (Hall et al., 2020; Schiff et al., 2018). STEPP gives patients the ability to continue accessing weekly behavioral health and SUD services including access to MOUD at our facility for up to 1 year postpartum. Involvement in STEPP provides several contact points for patients to obtain necessary clinical criteria and for the team to evaluate progress with HCV treatment. The barriers of performing laboratory assessments, liver imaging, obtaining childcare, and transportation proved to be large factors in patients being unable to complete treatment if they were not participating in STEPP. Integrated harm reduction models with HCV treatment appear to achieve higher rates of completion of HCV treatment, which may explain the higher success for STEPP patients in our cohort (Ziff et al., 2021).

Despite having a high prevalence of SUD and HCV, Ohio has been one of the slowest states to relax requirements for HCV treatment despite AASLD recommendations against these unnecessary requirements. The prohibitive cost of DAA treatment makes it difficult for patients to obtain treatment unless they comply with all Medicaid coverage requirements. When DAA treatments were first introduced, Ohio Medicaid required 6 months of documented sobriety before starting treatment, with multiple negative drug screens separated by at least a month (Davis, 2020). Imaging assessment for fibrosis was also required by all Medicaid plans until the end of 2021. This was a barrier that prevented several of our patients who had completed the necessary laboratory assessments from accessing treatment (Hepatitis C: State of Medicaid Access, 2022). The requirement for a hepatologist or infectious disease specialist to prescribe HCV treatment is no longer required, given the availability of several pangenotypic DAA treatments that are well tolerated. Once Ohio removed this requirement in February 2022, providers of any specialty may now prescribe DAA treatment (Hepatitis C: State of Medicaid Access, 2022). These changes in requirements should reduce barriers and make it easier for STEPP providers to directly prescribe treatment, and in turn, make treatment more accessible for patients. However, for patients without Medicaid or other insurance, access to HCV treatment continues to be difficult due to the high cost of these medications.

Telemedicine also played a large role in increasing the accessibility of treatment for our patients. Telemedicine has been shown to be effective for increasing treatment in several populations, including those with opioid use disorder (Beste et al., 2017; Morales-Arraez et al., 2021; Talal et al., 2019). Using telemedicine can reduce barriers to accessing care such as transportation, childcare, and connecting with providers specialized in HCV treatment. Telemedicine has been used successfully for postpartum depression, which suggests that it is a beneficial method of connecting with postpartum patients (Liu et al., 2022). Continued utilization of telemedicine may improve access to HCV treatment for this population.

Limitations to our program included the low overall number of patients diagnosed with HCV viremia at our institution during the short timeframe that the LTC model has been utilized. The overall low number of participants may have led to biased results due to under-enrollment. We suspect there were several HCV positive patients who were encountered on the labor and delivery unit or at another point who were not referred to our LTC team. In addition, our study is a single site study that was integrated into an existing co-located postpartum and SUD program, which may not be accessible at other institutions. Public health departments, especially those with obstetric services, could play an important role in providing HCV treatment for postpartum patients (Rosecrans et al., 2020). Public health providers could also assume the role of the infectious diseases department for hospitals where infectious disease or other subspecialists may not be easily accessible.

We hope to continue improving the identification and referral process for patients identified with HCV viremia while pregnant at our institution, especially those who were not enrolled in STEPP. Based on the gaps in our current LTC program, areas for improvement include increasing provider education, strengthening patient awareness of treatment eligibility requirements and identifying reliable methods of contacting patients.

In conclusion, our LTC model has greatly increased the number of postpartum patients treated for HCV in our co-located obstetric and SUD program. Utilizing telemedicine and partnerships with specialized care providers has proven beneficial in linking this underserved population to care.

### Supplementary Information

Below is the link to the electronic supplementary material.

Supplementary file1 (DOCX 269 kb)

## Data Availability

Data is available upon request from the corresponding author.
