# Supplementary figures and images for: Postpartum Hepatitis C Linkage to Care Program in a Co-located Substance Use Disorders Treatment Model
Source: Matern Child Health J. 2023 Sep 28;27(Suppl 1):87–93. doi: 10.1007/s10995-023-03770-w (PMC10691992; doi:10.1007/s10995-023-03770-w)

Supplemental Material: Patient handout regarding Hepatitis C treatment while postpartum


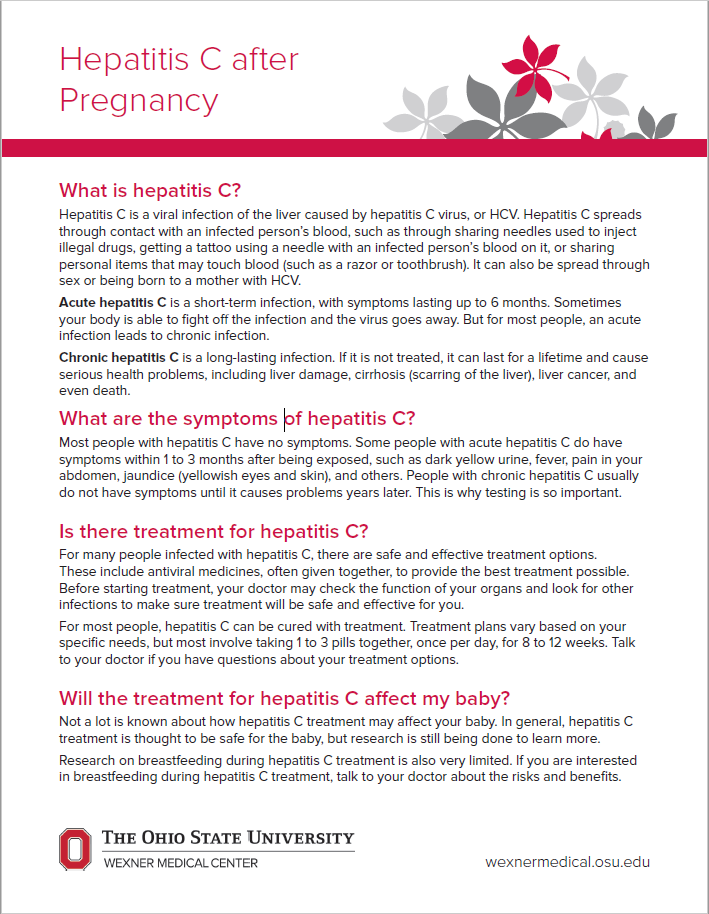


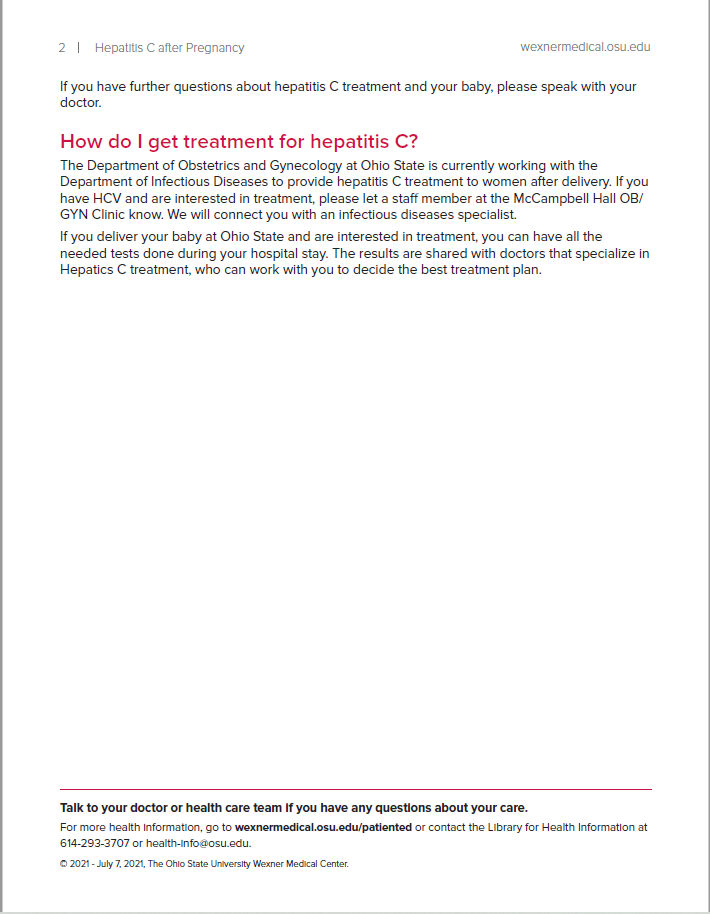

Supplement: Supplementary file 1 — Supplementary file1 (DOCX 269 kb) [file 10995_2023_3770_MOESM1_ESM.docx]
